# Inspiratory Muscle Training Improves Respiratory Muscle Strength and Cardiovascular Autonomic Regulation in Obese Young Men

**DOI:** 10.3390/life15081191

**Published:** 2025-07-27

**Authors:** Zhe Ren, Zeyu Zhou, Jikai Yang, Dongyue Wei, Hao Wu

**Affiliations:** 1 School of Kinesiology and Health, Capital University of Physical Education and Sports, Beijing 100191, China; renzhe0208@163.com (Z.R.); zzy687398@163.com (Z.Z.); 2 Comprehensive Key Laboratory of Sports Ability Evaluation and Research of the General Administration of Sport of China, Beijing 100191, China; 3 Beijing Key Laboratory of Sports Function Assessment and Technical Analysis, Beijing 100191, China; 4 School of Sports Management and Communication, Capital University of Physical Education and Sports, Beijing 100080, China; 24008046001@cupes.edu.cn; 5 College of Chinese Studies and Foreign Languages, Yantai Nanshan University, Yantai 265806, China; weidongyue2021@126.com

**Keywords:** inspiratory muscle training, respiratory muscle strength, cardiovascular autonomic regulation, heart rate variability, obesity

## Abstract

Objective: To investigate the effect of an 8-week inspiratory muscle training (IMT) intervention on respiratory muscle strength and cardiovascular autonomic regulation in obese young men. Methods: The study included 36 obese young men who met the inclusion and exclusion criteria. Participants were randomly divided into two groups: the IG (inspiratory muscle training group, n = 17), which underwent high-intensity IMT intervention for 8 weeks, 5 times a week, and the CG (control group, n = 18), which was not given any additional intervention. Assessed parameters included maximum inspiratory pressure (MIP), maximum expiratory pressure (MEP), systolic blood pressure (SBP), diastolic blood pressure (DBP), and heart rate (HR), as well as heart rate variability metrics such as the standard deviation of normal-to-normal intervals (SDNN), root mean square of successive differences (RMSSD), standard deviation of successive differences (SDSD), low-frequency power component (LF), high-frequency power component (HF), and LF/HF ratio. These measurements were taken both at baseline and following the completion of the 8-week intervention period. Results: After 8 weeks of IMT, the MIP and MEP of the IG increased by 31.8% and 26.5%, respectively (*p* < 0.01). In addition, SBP, DBP, and HR decreased by 2.2%, 3.2%, and 2.1%, respectively (*p* < 0.01). In the HRV time domain, SDNN and RMSSD increased by 54.1% and 33.5%, respectively (*p* < 0.01), and there was no significant improvement in SDSD (*p* > 0.05); in the HRV frequency domain, LF decreased by 40.5%, HF increased by 59.4% (*p* < 0.01), and the LF/HF ratio decreased by 58.2% (*p* < 0.05). Conclusion: An 8-week 80%MIP IMT intervention significantly improves respiratory muscle strength and cardiovascular autonomic regulation in obese young men, suggesting that IMT is a promising non-pharmacological strategy for mitigating obesity-related cardiovascular risk.

## 1. Introduction

Obesity has emerged as a pressing global public health challenge, with the prevalence of overweight and obesity in youth increasing over threefold since the late 20th century [1]. As a multifaceted risk factor for chronic diseases, obesity increases the risk of respiratory and cardiovascular disorders [2] and induces systemic pathophysiological changes. Specifically, excessive body weight and adiposity disrupt respiratory dynamics (e.g., chronic hypoxemia) by increasing chest wall mechanical loading, inducing respiratory muscle fatigue, and impairing lung ventilation efficiency [3]. Reduced respiratory muscle strength in obese people may lead to decreased respiratory efficiency and increase the risk of dyspnea and exercise intolerance, which further aggravate cardiovascular load and metabolic disorders [4]. Strengthening respiratory muscle strength has important clinical implications for obese people, as it not only improves respiratory function, but also reduces obesity-related cardiovascular risk by optimizing oxygenation efficiency and reducing cardiovascular stress [5].

Heart rate variability (HRV), a critical biomarker for cardiovascular risk assessment, has been widely used for early detection of acute and chronic cardiac disorders [6]. Obese individuals often exhibit autonomic dysfunction, characterized by reduced HRV and heightened sympathetic tone, which exacerbates cardiovascular risk through mechanisms such as vascular endothelial dysfunction and myocardial remodeling [7]. While aerobic exercise and dietary interventions reduce cardiovascular risk [8], adherence to these regimens remains a challenge, particularly among obese youth [9]. As an emerging non-pharmacological strategy, inspiratory muscle training (IMT) has gained attention for its potential to enhance respiratory muscle strength (e.g., diaphragm activation) and improve respiratory efficiency [10]. Unlike high-intensity aerobic training, IMT is a low-risk, venue-independent intervention that is adaptable to diverse populations, including obese individuals who struggle to sustain rigorous exercise [11]. IMT can strengthen respiratory muscles, reduce excessive activation of the inspiratory muscle metaboreflex, and decrease sympathetic nerve excitability [12,13,14]. This further increases HRV, improves autonomic balance, and reduces blood pressure by optimizing endothelial function and lowering peripheral vascular resistance [15,16].

Although evidence suggests that IMT enhances cardiovascular autonomic function in both general and clinical populations, there remains a lack of comprehensive studies examining its effects on cardiovascular autonomic nervous function specifically in obese individuals [5], with research focusing on obese adolescents being particularly limited. Consequently, the present study was designed to assess the impact of an 8-week high-intensity IMT intervention on respiratory muscle strength, blood pressure, and heart rate variability among obese young men. The findings will offer evidence-based insights into developing non-pharmacological strategies to mitigate obesity-related cardiovascular risks and will guide the design of tailored exercise programs for this population.

## 2. Materials and Methods

### 2.1. Participants

A total of 60 male college students were recruited as volunteers from multiple universities in Beijing, with recruitment commencing on 10 October 2022. Following screening, 36 obese male college students were enrolled. All participants provided written informed consent prior to screening and were thoroughly informed about the experimental procedures. This study strictly adhered to the ethical standards outlined in the Declaration of Helsinki and was approved by the Research Ethics Committee of the Capital University of Physical Education and Sports (approval number: 2022A58).

Inclusion criteria included the following: (1) an age of 18–25 years old; (2) body mass index (BMI) ≥ 28 kg/m^2^ [17]; (3) no history of regular exercise; and (4) voluntary cooperation with the inspiratory muscle training experiment. Exclusion criteria were (1) an inability to complete the 8-week intervention; (2) severe cardiovascular disease; (3) concurrent participation in other interventions affecting cardiovascular or respiratory outcomes; and (4) pre-existing health conditions that contraindicate IMT.

### 2.2. Experimental Design

This study was conducted and reported in accordance with the CONSORT guidelines [18] to ensure transparency and completeness in reporting. A single-blind randomized controlled trial was employed to evaluate the efficacy of an 8-week IMT intervention in obese young men. Thirty-six eligible participants were randomly assigned (1:1 ratio) to either the IMT group (IG, n = 18) or the control group (CG, n = 18) using a computer-generated randomization list, stratified by baseline maximum inspiratory pressure (MIP) to ensure balanced distribution of respiratory muscle strength. Allocation concealment was achieved through sealed opaque envelopes managed by an independent statistician. The IG received an IMT regimen with a high-intensity 80%MIP. The CG maintained their usual lifestyle without any structured intervention.

Baseline assessments, conducted prior to randomization, included anthropometric measurements (height, weight, and BMI), medical history screening, and self-reported physical activity levels assessed using the International Physical Activity Questionnaire. Outcome measures, collected at both baseline and post-intervention, comprised respiratory muscle strength, blood pressure, resting heart rate, and HRV. To minimize detection bias, outcome evaluations were performed by trained researchers who were blinded to both the intervention and randomization processes.

### 2.3. Inspiratory Muscle Training Program

The IMT intervention utilized an intelligent breathing trainer (Xeek BW05, Xiamen, China) over an 8-week period [19]. IG participants completed five 15 min sessions per week, with each session comprising two sets of 30 maximal inspiratory maneuvers separated by 3 min rest intervals. Training intensity was individually tailored to 80% of baseline MIP, measured using the same device with participants seated, adhering to standardized protocols. This regimen has been shown to not only strengthen respiratory muscles but also enhance overall functional capacity, improve quality of life, and positively affect cardiovascular health with a high degree of safety [20].

Participants completed a 30 min orientation session to learn proper technique, resolve technical issues, and promote adherence. Training compliance was tracked using device-logged data (session duration, pressure, and flow metrics), requiring at least 80% completion for inclusion in the analyses. Safety was monitored via training diaries, where participants noted adverse events such as dizziness or respiratory discomfort, with weekly device calibration conducted per manufacturer protocols. Blinded researchers performed all baseline and post-intervention assessments.

### 2.4. Respiratory Muscle Strength Test

MIP and maximal expiratory pressure (MEP) were measured using a XeeK BW05 device equipped with a biofeedback module to monitor nasal/oral airflow and thoracoabdominal movement, ensuring standardized maneuvers. Participants wore a nose clip to prevent nasal leakage. For MIP, participants performed a rapid, forceful inspiration from residual volume. For MEP, participants executed a rapid, forceful expiration from total lung capacity following tidal breathing. All measurements were conducted in a standing position [10].

According to ATS/ERS standards, each participant completed three acceptable maneuvers for both MIP and MEP, with a plateau pressure sustained for ≥1 s. Additional maneuvers were performed only when necessary to achieve reproducibility (highest values within 10% variability). The highest value was recorded as the final MIP/MEP. Test-retest reliability was assessed using intra-class correlation coefficients (ICC > 0.90 for MIP and MEP).

### 2.5. Heart Rate and Blood Pressure Test

Systolic blood pressure (SBP), diastolic blood pressure (DBP), and resting heart rate (HR) were measured in the morning using a calibrated oscillometric blood pressure monitor (Omron U12, Kyoto, Japan) equipped with a HEM-CR24 cuff (arm circumference: 22–32 cm). The device was calibrated against a mercury sphygmomanometer prior to the study according to ANSI/AAMI/ISO 81060-2:2018. [21] protocols, ensuring measurement differences of ≤5 mmHg. Mid-upper arm circumference was measured to confirm the suitability of the HEM-CR24 cuff, with bladder width and length covering approximately 40% and 80% of the arm circumference, respectively, in accordance with ATS/ERS guidelines, ensuring a cuff–arm size mismatch of less than 2 cm.

To minimize measurement errors, participants were asked to sit quietly for at least 5 min in a quiet and comfortable environment prior to each blood pressure assessment. Measurements were conducted in the morning, with participants maintaining an upright seated position and their left arm supported at heart level. Three readings were obtained at 1 to 2 min intervals, and the average of these three values was used as the final blood pressure and heart rate result. If the difference between the highest and lowest readings exceeded 5 mmHg, a fourth measurement was performed and the average was recalculated accordingly.

### 2.6. Heart Rate Variability Test

Heart rate variability (HRV) was evaluated using a heart rate variability analysis system (Omegawave Sport Technology System, Espoo, Finland) [22]. Continuous ECG signals were recorded during a morning resting state, and autonomic nervous function was assessed through both time-domain and frequency-domain analyses. The test strictly conformed to international HRV testing standards and was conducted in a controlled environment (temperature: 22 ± 1 °C; relative humidity: 50 ± 5%; ambient noise level: <40 dB). Participants were positioned supine, and five-minute ECG recordings were obtained following a ten-minute acclimatization period. Data analysis utilized the Omegawave Sport Technology System, which incorporates an automatic artifact correction algorithm based on threshold detection and cubic spline interpolation. The proportion of R-R intervals requiring interpolation across all participants was less than 5%, thereby ensuring high data quality.

The indicators selected for the time-domain analysis included the following: the standard deviation of normal sinus RR intervals (SDNN), which reflects overall heart rate variability (HRV); the root mean square of successive differences between adjacent RR intervals (RMSSD), indicative of parasympathetic activity; and the standard deviation of differences between adjacent RR intervals (SDSD), representing parasympathetic regulatory capacity. RMSSD also serves as a measure of parasympathetic regulation based on the analysis of consecutive normal RR interval differences. Frequency-domain analysis was conducted using standard spectral bands: the low-frequency band (LF, 0.04–0.15 Hz), which reflects both sympathetic and parasympathetic activity, and the high-frequency band (HF, 0.15–0.40 Hz), primarily reflecting parasympathetic activity. The LF/HF ratio was calculated to evaluate sympathovagal balance.

### 2.7. Data and Statistical Analysis

Results are reported as mean ± standard deviation (M ± SD). Independent samples *t*-tests were conducted to compare baseline characteristics of the participants. To evaluate differences in respiratory muscle strength, cardiovascular autonomic function, and HRV between groups at baseline and following an 8-week intervention period, a 2 × 2 (group × time) repeated-measures analysis of variance (ANOVA) was used. Effect sizes were calculated to quantify the magnitude of between-group differences, with partial eta squared (*η*^2^) serving as the effect size measure for the repeated-measures ANOVA. Effect sizes were interpreted according to established thresholds: small (*η*^2^ = 0.01), medium (*η*^2^ = 0.06), and large (*η*^2^ = 0.14). Statistical significance was set at *p* < 0.05. All statistical analyses were performed using SPSS software (version 27.0) and GraphPad Prism (version 9.5).

## 3. Results

**Figure 1:**
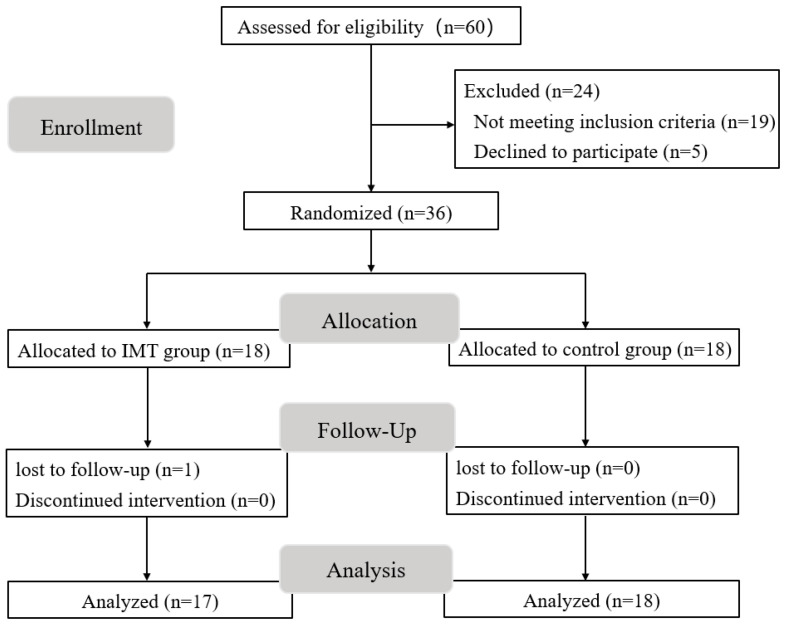
Flow chart of participants.

The flow diagram is presented in Figure 1. Twenty-four participants were excluded from the study. After randomization (n = 36), one participant in the IG withdrew from the study. A total of 17 participants in the IG and 18 participants in the CG were included in the statistical analysis. All included participants completed the entire program and did not experience any adverse events throughout the training program or during the evaluation of both groups. The baseline characteristics of the participants are summarized in Table 1. The analysis revealed no significant differences in baseline values between the IG and CG prior to the intervention (*p* < 0.05), thus meeting the requirements of this study.

### 3.1. Respiratory Muscle Strength

**Figure 2:**
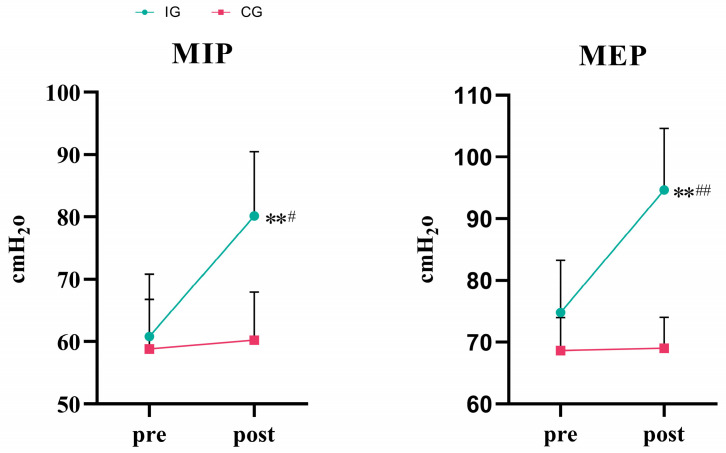
Graph of changes in MIP and MEP indicators. ** *p* < 0.01 vs. baseline; ^#^ *p* < 0.05 vs. control group; ^##^ *p* < 0.01 vs. control group.

As shown in Table 2 and Figure 2, a two-way ANOVA showed that the effect of group × time on MIP was statistically significant (*F* = 7.859, *p* = 0.012, *η*^2^ = 0.304). The simple main effect analysis showed that there was no significant difference in MIP between the two groups (*p* = 0.062). However, the effect of time on MIP was not statistically significant (*p* = 0.370). The MIP in the IG significantly increased (*p* < 0.01). Compared with the CG, the difference was statistically significant after 8 weeks of training (*p* < 0.05).

Two-way ANOVA showed that the effect of group × time on MEP was statistically significant (*F* = 95.833, *p* < 0.001, *η*^2^ = 0.842). The simple main effect analysis showed that there was a significant difference in MEP between the two groups (*p* < 0.001). The simple main effect analysis showed that the effect of time on MEP was statistically significant (*p* < 0.001). The MEP in the IG significantly increased (*p* < 0.01). Compared with the CG, the difference was statistically significant after 8 weeks of training (*p* < 0.01).

### 3.2. Cardiovascular Function

**Figure 3:**
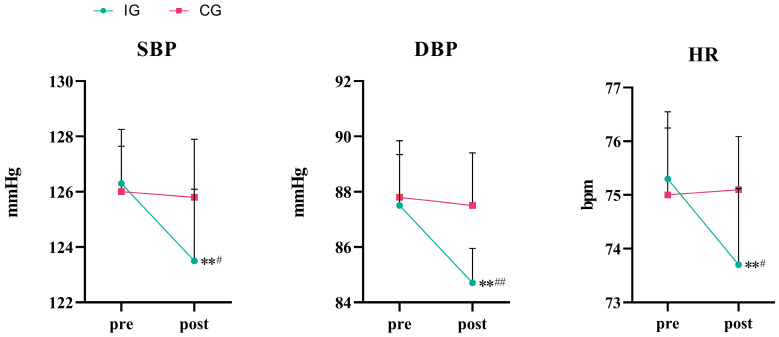
Plot of changes in SBP, DBP, and HR indicators.** *p* < 0.01 vs. baseline; ^#^ *p* < 0.05 vs. control group; ^##^ *p* < 0.01 vs. control group.

As shown in Table 3 and Figure 3, a two-way analysis of variance showed that the influence of group × time on SBP was statistically significant (*F* = 12.366, *p* = 0.002, *η*^2^ = 0.407). A simple main effect analysis showed that there was no significant difference in SBP between the two groups (*p* = 0.268). The effect of time on SBP was also statistically significant (*p* = 0.001). The SBP in the IG significantly decreased (*p* < 0.01). Compared with the CG, the difference was statistically significant after 8 weeks of training (*p* < 0.05).

The effect of group × time on DBP was statistically significant (*F* = 18.939, *p* < 0.001, *η*^2^ = 0.513). A simple main effect analysis showed that there was no significant difference in DBP between the two groups (*p* = 0.052). The effect of time on DBP was also statistically significant (*p* ≤ 0.001). The DBP in the IG significantly decreased (*p* < 0.01). Compared with the CG, the difference was statistically significant after 8 weeks of training (*p* < 0.01).

Similarly, the effects of group × time on resting HR were statistically significant (*F* = 17.000, *p* = 0.001, *η*^2^ = 0.486). A simple main effect analysis showed that there was no significant difference in HR between the two groups (*p* = 0.298). The effect of time on HR was also statistically significant (*p* = 0.002). The HR in the IG significantly decreased (*p* < 0.01). Compared with the CG, the difference was statistically significant after 8 weeks of training (*p* < 0.05).

### 3.3. Autonomic Function

**Figure 4:**
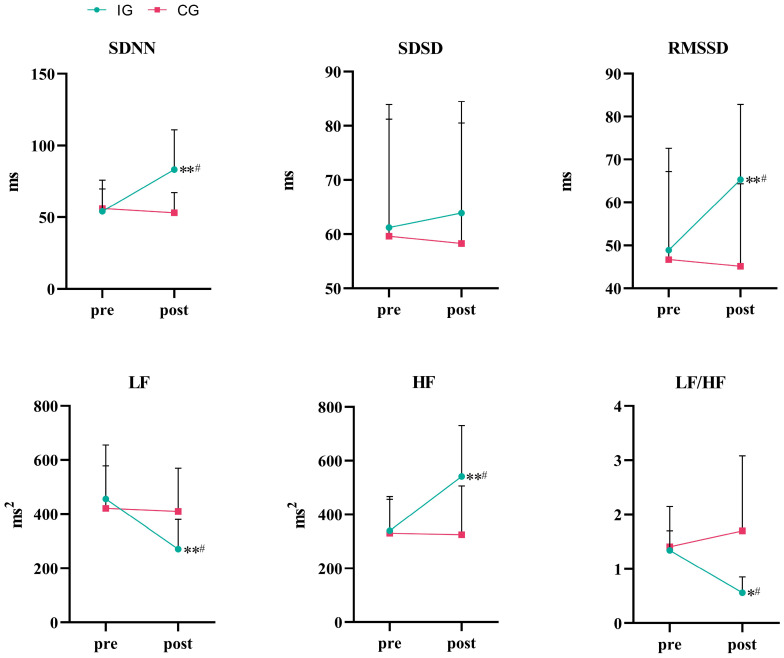
Variation in HRV metrics in time and frequency domains. * *p* < 0.05 vs. baseline; ** *p* < 0.01 vs. baseline; ^#^ *p* < 0.05 vs. control group.

Regarding the HRV time domain indexes shown in Table 4 and Figure 4, a two-way analysis of variance showed that the effect of group × time on SDNN was statistically significant (*F* = 6.652, *p* = 0.019, *η*^2^ = 0.270). The simple main effect analysis showed no significant difference in SDNN between the two groups (*p* = 0.120). The effect of time on SDNN was statistically significant (*p* = 0.049). The SDNN in the IG significantly increased (*p* < 0.01). Compared with the CG, the difference was statistically significant after 8 weeks of training (*p* < 0.05). The effect of group × time on SDSD was not statistically significant (*F* = 0.214, *p* = 0.649, *η*^2^ = 0.012). The effect of group × time on RMSSD was statistically significant (*F* = 20.466, *p* < 0.001, *η*^2^ = 0.532). Simple main effect analysis showed that there was no significant difference in RMSSD between the two groups (*p* = 0.224). The effect of time on RMSSD was statistically significant (*p* = 0.002). The RMSSD in the IG significantly increased (*p* < 0.01). Compared with the CG, the difference was statistically significant after 8 weeks of training (*p* < 0.05).

A two-way ANOVA also showed that the effect of group × time on LF was statistically significant (*F* = 5.367, *p* = 0.033, *η*^2^ = 0.230). A simple main effect analysis showed that there was no significant difference in LF between the two groups (*p* = 0.403). The effect of time on LF was statistically significant (*p* = 0.017). The LF in the IG significantly decreased (*p* < 0.01). Compared with the CG, the difference was statistically significant after 8 weeks of training (*p* < 0.05). The effect of group × time on HF was statistically significant (*F* = 7.204, *p* = 0.015, *η*^2^ = 0.286). A simple main effect analysis showed that there was no significant difference in HF between the two groups (*p* = 0.081). The effect of time on HF was statistically significant (*p* = 0.021). The HF in the IG significantly increased (*p* < 0.01). Compared with the CG, the difference was statistically significant after 8 weeks of training (*p < 0.05).* The effect of group × time on the LF/HF ratio was statistically significant (*F* = 5.273, *p* = 0.034, *η*^2^ = 0.227). The simple main effect analysis showed that there was a significant difference between the two groups (*p* = 0.044). The effect of time on LF/HF was not statistically significant (*p* = 0.306). The LF/HF ratio in the IG significantly decreased (*p* < 0.05). Compared with the CG, the difference was statistically significant after 8 weeks of training (*p* < 0.05).

## 4. Discussion

### 4.1. Effect of 8 Weeks of IMT on Respiratory Muscle Strength

Due to the long-term sedentary lifestyle of obese youth groups, their respiratory muscle groups show typical functional degradation resulting from insufficient stimulation [4]. Weight gain further exacerbates the burden on respiratory muscles, especially during physical activity, and the imbalance between impaired respiratory muscle function and metabolic demand often induces respiratory compensatory phenomena. IMT is beneficial for the health of obese individuals. By enhancing the strength of respiratory muscles, IMT can increase respiratory capacity, promote muscle oxygenation, reduce respiratory muscle fatigue, alleviate breathing difficulties, improve respiratory efficiency, and enhance performance during physical activities [5]. This study found that an 8-week IMT intervention significantly enhanced MIP and MEP in obese young men.

Respiratory muscles are skeletal muscles in form and function, and their response to training follows the physiological properties of skeletal muscles [23]. Studies have also shown that IMT significantly enhances the strength and endurance of respiratory muscles in sedentary college students and reduces dyspnea [24]. Ref. [25] highlighted the translational potential of high-resistance inspiratory muscle strength training. Enright et al. [26] showed, in a study of healthy people, that 8 weeks of IMT not only significantly increased MIP and MEP, but also significantly improved the overall respiratory function by increasing the thickness of the diaphragm and optimizing the efficiency of pulmonary ventilation. This finding suggests that IMT has similarly significant respiratory function benefits in the non-obese population [26]. Lee et al. also found that appropriate IMT was able to increase respiratory muscle thickness and improve respiratory muscle strength, which is consistent with the present study [27]. IMT can improve maximal inspiratory pressure in healthy young men [28]. These results suggest that successful use in healthy populations provides indirect evidence for obese populations that IMT is both structurally and functionally adapted to respiratory muscle strength. Increases in MIP and MEP reflect positive physiological adaptations attributed to controlled training supervision and accurate measurement protocols.

### 4.2. Effects of 8-Week IMT on Cardiovascular Function

The normal blood pressure range for SBP is 90–120 mmHg, while the normal range for DBP is 60–80 mmHg [29]. The prevalence of blood pressure values close to the hypertensive threshold in the obese youth population is closely associated with obesity-related hormonal regulation disorders, fluid imbalance, and increased cardiovascular load, and these pathophysiologic changes can increase peripheral arterial resistance and significantly increase the risk of cardiovascular disease [30]. In the present study, we found that all of the cardiovascular functions of obese young men significantly improved after 8 weeks of IMT intervention, including a significant decrease in resting HR, and a decrease in SBP and DBP by 2.21% and 3.2%, respectively.

IMT plays a pivotal role in the improvement of cardiovascular health as a non-pharmacological intervention to control respiratory patterns and slow down respiratory rate [31]. IMT alters respiratory patterns, resulting in a slower respiratory rate and increased breathing depth, which directly influences cardiovascular regulation. During the inspiratory phase, thoracic cavity expansion reduces venous return, leading to decreased cardiac output and myocardial contractility, thereby lowering blood pressure [32]. Moreover, the choice of training load affects the intervention’s effectiveness, with Ferreira et al. demonstrating a dose–response relationship between the cardiovascular benefits of IMT and changes in breathing pattern and load intensity [15]. Resisted IMT enhances breathing pattern adaptation and modulates reflex mechanisms [33]. In a meta-analysis, IMT with loading resistance was found to be effective in reducing SBP and DBP, whereas training without loading resistance only improved DBP [3]. This supports the load selection strategy of the present study. DeLucia showed a significant decrease in both systolic and diastolic blood pressure after a 6-week IMT training intervention with 30% MIP in sedentary college students [34]. The decrease in DBP, a sensitive indicator of peripheral vascular resistance, suggests that IMT may enhance vascular tone by modulating autonomic homeostasis. Greater peripheral vascular resistance results in higher DBP, as the heart must overcome increased resistance to eject blood [35]. Bersten et al.’s study of IMT in patients with heart failure showed that pulmonary artery compression reduced pulmonary blood flow and increased systemic vascular resistance, but IMT reduced cardiac output by increasing intrathoracic pressure, resulting in decreased ventricular filling and a significant reduction in DBP [36]. In addition, Salvadego et al. performed intermittent IMT for 3 weeks in obese adolescents and showed that the training significantly reduced resting HR and respiratory discomfort and improved exercise tolerance in the subjects [37]. This favorable modulation of heart rate may be related to the improvement of vagal tone and reduction of sympathetic excitability by IMT.

### 4.3. Effects of 8-Week IMT on Autonomic Function

HRV, a key physiological indicator for assessing the dynamic homeostasis of the cardiac autonomic nervous system (ANS), is significantly correlated with cardiac adaptations, coronary artery elasticity, and systemic inflammatory status [38]. Elevated HRV indicates improved cardiovascular regulation, whereas reduced HRV is strongly associated with increased cardiac load, immune disorders, and elevated risk of arrhythmias [39]. Maintaining or increasing HRV not only prevents cardiovascular disease, but also provides an important reference for cardiovascular health management [40]. Obesity-induced ANS imbalance is often characterized by sympathetic overactivation and reduced vagal tone, which may contribute to low HRV [41].

Eight weeks of IMT significantly improved heart rate variability (HRV). In the time domain, SDNN and RMSSD increased significantly, while SDSD showed no significant change. In the frequency domain, HF increased significantly, LF decreased significantly, and the LF/HF ratio decreased significantly. These changes indicate that IMT significantly enhances sympathetic–parasympathetic balance by reducing sympathetic activity and increasing parasympathetic modulation, providing new evidence for non-pharmacological interventions targeting obesity-related autonomic dysfunction. Furthermore, recent studies have found another mechanism by which IMT may reduce cortisol levels and cortisol/testosterone ratio and enhance autonomic regulation by regulating the hypothalamic–pituitary–adrenal axis, thereby supplementing its effect on HRV [42].

The improvement in autonomic function following 8 weeks of high-intensity IMT may be mediated through multiple physiological mechanisms. Inspiratory muscle weakness frequently causes fatigue, leading to metabolite accumulation in the muscles. This accumulation stimulates unmyelinated somatic afferent fibers, eliciting sympathetic reflex activity and peripheral vasoconstriction at rest, a phenomenon termed the “inspiratory muscle metaboreflex” [13]. Inspiratory muscle fatigue has been shown to increase activation of the metaboreflex, thereby elevating peripheral sympathetic nerve activity [43]. The delay in the activation of the metabolic reflex in the inspiratory muscles while performing physical activity is a protective reflex that redistributes blood from the peripheral region to the respiratory muscle region, raising the threshold for the metabolic reflex [44] and preventing dyspnea due to insufficient respiratory muscle blood supply [16]. Increased respiratory muscle strength following IMT may delay the onset of this reflex, promoting a more active lifestyle, enhancing fatigue resistance, and reducing LF power, thereby mitigating sympathetic overactivity. The deep, slow breathing pattern developed during training may directly enhance parasympathetic modulation of the heart via the pulmonary traction receptor–brainstem vagal pathway [45]. Consistent with previous studies, we also observed an increase in HF and RMSSD indexes regarding HRV and an increase in parasympathetic modulation after 8 weeks of IMT, consistent with previous studies. Jaenisch showed that 6 weeks of IMT intervention resulted in an improvement of cardiac autonomic function in rats, which was manifested by a decrease in the absolute value of the LF spectra and the indexes of sympathetic modulation, while a significant increase in the HF spectra and RMSSD was observed [46]. From a clinical perspective, IMT improves cardiac autonomic tone and systemic neuromodulation [47], which is of great significance for obese young men aged 18–25 years. Given the elevated cardiovascular risk associated with autonomic imbalance, enhanced HRV may decrease the incidence of arrhythmias and other cardiovascular complications. IMT offers a promising and effective non-pharmacological strategy for managing cardiovascular health in individuals with obesity.

### 4.4. Limitations of the Study

This study has several limitations. The inclusion of only male participants limits the generalizability of the findings to women. Monitoring of lifestyle factors, such as diet and physical activity, requires greater rigor. The 8-week duration of the intervention limits insights into longer-term effects. Although significant blood pressure reductions were observed, the small magnitude of these reductions may exaggerate their clinical significance. Additionally, the inspiratory muscle metaboreflex pathway was not assessed. Future research should focus on strictly monitoring lifestyle factors, investigating the dose–response effects of IMT in diverse populations, exploring the synergistic effects of IMT combined with other exercise modalities, and conducting long-term follow-up studies after IMT interventions. This study provides a detailed examination of the potential benefits of IMT in regulating cardiovascular autonomic function in obese young men, healthy individuals, athletes, women, and the elderly from multiple perspectives.

## 5. Conclusions

An 8-week high-intensity IMT intervention significantly enhances respiratory muscle strength and cardiovascular autonomic regulation in obese young men. Specifically, IMT increases MIP and MEP, while significantly reducing SBP, DBP, and resting HR. HRV was also significantly improved, with increases in SDNN, RMSSD, and HF and decreases in LF and the LF/HF ratio. These findings demonstrate the effectiveness of IMT as a nonpharmacological intervention in reducing obesity-related cardiovascular risk. A minimum of 8 weeks of IMT with regular monitoring and evaluation is recommended to achieve these benefits.

## Figures and Tables

**Table 1:** Subjects’ basic information (M ± SD).

Group	IG (n = 17)	CG (n = 18)	*p*
Age (years)	21.1 ± 1.79	21.0 ± 2.06	0.909
Height (cm)	175.31 ± 4.48	176.48 ± 5.15	0.524
Weight (kg)	90.10 ± 5.82	89.50 ± 5.14	0.810
BMI (kg/m^2^)	29.3 ± 0.96	28.7 ± 1.00	0.219
MIP (cmH_2_O)	60.83 ± 9.98	58.82 ± 7.96	0.624
MEP (cmH_2_O)	74.81 ± 8.43	68.63 ± 5.33	0.066
SBP (mmHg)	126.30 ± 1.34	126.00 ± 2.26	0.722
DBP (mmHg)	87.50 ± 1.84	87.80 ± 2.04	0.734
HR (bpm)	75.30 ± 1.25	75.00 ± 1.25	0.598

**Table 2:** Changes in respiratory muscle strength indexes (M ± SD).

Variables	Group	Pre	Post	Mean Difference (95% CI)	*p* Value
MIP (cmH_2_O)	IG (n = 17)	60.83 ± 9.98	80.16 ± 10.30 **^#^	−19.33[−28.82,−9.85]	<0.001
	CG (n = 18)	58.82 ± 7.96	60.24 ± 7.69	−1.42[−8.06,10.92]	0.755
MEP (cmH_2_O)	IG (n = 17)	74.81 ± 8.43	94.63 ± 10.01 **^##^	−19.82[−22.77,−16.87]	<0.001
	CG (n = 18)	68.63 ± 5.33	69.01 ± 5.01	−0.38[−3.33,2.58]	0.792

** *p* < 0.01 vs. baseline; ^#^ *p* < 0.05 vs. control group; ^##^ *p* < 0.01 vs. control group.

**Table 3:** Changes in SBP, DBP, and HR index indicators (M ± SD).

Variables	Group	Pre	Post	Mean Difference (95% CI)	*p* Value
SBP (mmHg)	IG (n = 17)	126.30 ± 1.34	123.50 ± 2.59 **^#^	2.80[1.70,3.99]	<0.001
	CG (n = 18)	126.00 ± 2.26	125.80 ± 2.10	0.20[−0.90,1.30]	0.707
DBP (mmHg)	IG (n = 17)	87.50 ± 1.84	84.70 ± 1.25 **^##^	2.80[1.95,3.65]	<0.001
	CG (n = 18)	87.80 ± 2.04	87.50 ± 1.90	0.30[−0.55,1.15]	0.470
HR	IG (n = 17)	75.30 ± 1.25	73.70 ± 1.42 **^#^	1.60[0.99,2.21]	<0.001
(bpm)	CG (n = 18)	75.00 ± 1.25	75.10 ± 0.99	−0.10[0.74,−0.71]	0.736

** *p* < 0.01 vs. baseline; ^#^ *p* < 0.05 vs. control group; ^##^ *p* < 0.01 vs. control group.

**Table 4:** Changes in HRV time and frequency domain metrics ($\bar{x}\pm s$).

Variables	Group	Pre	Post	Mean Difference (95% CI)	*p* Value
SDNN (ms)	IG (n = 17)	54.00 ± 15.69	83.20 ± 27.73 **^#^	−29.20[−47.69,−10.71]	0.004
	CG (n = 18)	56.00 ± 19.78	53.10 ± 14.08	2.90[−15.59,21.39]	0.746
SDSD (ms)	IG (n = 17)	61.20 ± 22.70	63.90 ± 20.57	−2.70[−15.54,10.14]	0.664
	CG (n = 18)	59.60 ± 21.60	58.30 ± 22.23	1.30[−11.54,14.14]	0.834
RMSSD (ms)	IG (n = 17)	48.90 ± 23.73	65.30 ± 17.51 **^#^	−16.40[−22.31,−10.49]	<0.001
	CG (n = 18)	46.70 ± 20.48	45.10 ± 19.27	−1.60[−7.51,4.31]	0.577
LF (ms^2^)	IG (n = 17)	455.40 ± 199.87	270.80 ± 109.77 **^#^	184.60[73.53,295.66]	0.003
	CG (n = 18)	420.80 ± 157.00	409.40 ± 159.63	11.40[−99.67,122.47]	0.832
HF (ms^2^)	IG (n = 17)	339.50 ± 116.89	541.10 ± 188.95 **^#^	−201.60[−316.28,−86.92]	0.002
	CG (n = 18)	330.50 ± 135.94	324.90 ± 189.72	5.60[−1.9.08,120.28]	0.919
LF/HF	IG (n = 17)	1.34 ± 0.36	0.56 ± 0.29 *^#^	0.78[0.09,1.47]	0.029
	CG (n = 18)	1.41 ± 0.74	1.70 ± 1.38	−2.90[−0.98,0.40]	0.33

* *p* < 0.05 vs. baseline; ** *p* < 0.01 vs. baseline; ^#^ *p* < 0.05 vs. control group.

## Data Availability

The data that support the findings of this study are available on request from the corresponding author.
